# Reproductive competition triggers mass eviction in cooperative banded mongooses

**DOI:** 10.1098/rspb.2015.2607

**Published:** 2016-03-16

**Authors:** Faye J. Thompson, Harry H. Marshall, Jennifer L. Sanderson, Emma I. K. Vitikainen, Hazel J. Nichols, Jason S. Gilchrist, Andrew J. Young, Sarah J. Hodge, Michael A. Cant

**Affiliations:** 1 Centre for Ecology and Conservation, University of Exeter, Penryn Campus, Penryn, Cornwall TR10 9FE, UK; 2 School of Natural Sciences and Psychology, Liverpool John Moores University, Liverpool L3 3AF, UK; 3 School of Life Sciences, Napier University, Edinburgh EH10 5DT, UK

**Keywords:** eviction, conflict, cooperation, reproductive competition, coercion, forced dispersal

## Abstract

In many vertebrate societies, forced eviction of group members is an important determinant of population structure, but little is known about what triggers eviction. Three main explanations are: (i) the reproductive competition hypothesis, (ii) the coercion of cooperation hypothesis, and (iii) the adaptive forced dispersal hypothesis. The last hypothesis proposes that dominant individuals use eviction as an adaptive strategy to propagate copies of their alleles through a highly structured population. We tested these hypotheses as explanations for eviction in cooperatively breeding banded mongooses (*Mungos mungo*), using a 16-year dataset on life history, behaviour and relatedness. In this species, groups of females, or mixed-sex groups, are periodically evicted *en masse*. Our evidence suggests that reproductive competition is the main ultimate trigger for eviction for both sexes. We find little evidence that mass eviction is used to coerce helping, or as a mechanism to force dispersal of relatives into the population. Eviction of females changes the landscape of reproductive competition for remaining males, which may explain why males are evicted alongside females. Our results show that the consequences of resolving within-group conflict resonate through groups and populations to affect population structure, with important implications for social evolution.

## 1. Introduction

Individuals living in ‘viscous’ groups, in which there are severe constraints on dispersal, face numerous conflicts of interest with other group members. In cooperative breeders, conflict can arise over reproduction, helping effort, parental care and dispersal [1–3]. Much theoretical and empirical work has focused on how individuals resolve these within-group conflicts. In both insect and vertebrate societies, individuals may use threats, aggression, punishment and various strategies of negotiation to settle conflicts without breaking up the group [4–6]. In other cases, however, within-group conflict results in the forcible eviction of one or more group members, typically following intense, targeted aggression [7–10]. Eviction often leads to the permanent dispersal of individuals, or coalitions of individuals, and may be a major source of gene flow between groups [11,12]. Determining what triggers eviction is therefore important to understand the factors that shape population genetic structure and demography in viscous populations, and hence social evolution [13,14].

In social vertebrates, eviction often appears to be driven by conflict over reproductive or social status within groups. In some mammal species, dominant individuals maintain their reproductive monopoly by evicting reproductive competitors from the group [7,15]. For example, in meerkats, *Suricata suricatta*, dominant females evict subordinate females in the latter half of their (own) pregnancy, often as a strategic measure to avoid infanticidal attacks on their pups [16]. Subordinates that are pregnant when evicted experience a deterioration in condition, elevated stress levels, and often spontaneously abort before gaining readmittance to their group [7]. Consequently, eviction reduces future, as well as current, reproductive competition from the perspective of the dominant by suppressing subordinates' future reproductive success. In fishes that form size-based hierarchies, dominant individuals use the threat of eviction to deter subordinates from growing large enough to challenge their position [17–19]. As a result, in the coral dwelling goby, *Paragobiodon xanthosomus*, subordinates starve themselves to avoid triggering eviction [20].

Alternative explanations for eviction are based on the idea that dominant individuals can use eviction to coerce their subordinates to help. For example, the pay-to-stay hypothesis [21] suggests that dominant individuals can threaten helpers with eviction unless they behave cooperatively. Additionally, dominant individuals might evict temporarily to coerce helpers to work harder on their return [22], or evict permanently to establish a reputation for punishment and thereby induce remaining helpers to cooperate [23]. Clear evidence in support of such coercive mechanisms comes from the cooperative cichlid, *Neolamprologus pulcher*. Helpers that are experimentally prevented from helping are subject to elevated aggression from dominants and subsequently help more, as predicted if aggression is a signal of impending eviction [9,24]. In addition, helpers that are temporarily removed are often evicted on their return, and those that are reaccepted work harder thereafter [25]. In cooperative birds and mammals, evidence for the pay-to-stay hypothesis is less clear-cut. In superb fairy-wrens, *Malurus cyaneus*, temporary removal of helpers results in increased aggression from dominants [26], while in naked mole-rats, *Heterocephalus glaber*, and meerkats there is evidence that uncooperative helpers are subject to aggression from dominant breeders [27,28]. In addition, temporarily evicted female meerkats are more likely to allolactate on their return to the group than non-evicted females [29]. By contrast, studies of bell miners, *Manorina melanophrys* [30,31] and chestnut-crowned babblers, *Pomatostomus ruficep* [32] have failed to find support for mechanisms based on pay-to-stay or punishment.

A third, unexplored hypothesis is that eviction is an adaptive forced dispersal strategy used by breeders to spread copies of their alleles through the wider population. Traditionally, studies of cooperative breeders have used the number of surviving offspring as a measure of fitness. However, groups of cooperative breeders can be thought of as miniature populations embedded within a wider metapopulation [33]. In this kind of structured population, what matters is not just the number of offspring that are successfully raised, but how successful these offspring are at dispersing to form or join new groups, and in turn produce dispersing offspring of their own—sometimes referred to as metapopulation fitness [34,35]. Forced dispersal could be a strategy to maximize metapopulation fitness, over and above any immediate benefits evictors might gain by reducing local competition (although more intense local competition should strengthen selection for forced dispersal). If eviction is primarily a strategy to export copies of alleles, one would expect dominants to evict related individuals rather than unrelated individuals, to evict when local competition is high, and to evict when the evictees have the best chance of dispersing successfully to found or usurp new groups.

Banded mongooses, *Mungos mungo*, are a good system to test hypotheses about the causes and function of eviction in cooperative societies because evictions are common and conspicuous. This species lives in mixed-sex groups of around 20 adults, plus offspring. Each eviction event starts suddenly, lasts several days, and involves intense aggression from males and females directed towards multiple individuals. Aggression continues until groups of females, and on occasion groups of males alongside them, are driven away from the group, sometimes limping or bleeding [8] (see the video of a typical eviction event in the electronic supplementary material). Up to 26 individuals have been observed to be evicted in a single eviction event [8]. Evictees are sometimes allowed to return to their group within a week (‘temporary evictions’) or they may disperse permanently (‘permanent evictions'; [36]). In mixed-sex, permanent eviction events, males and females form same-sex cohorts and disperse separately, most likely to avoid inbreeding [37].

In banded mongoose groups, there is intense reproductive competition among both males and females [38]. Among males, a few high-ranking ‘mate guarding’ males aggressively monopolize access to females during oestrus: on average, the oldest three males sire 85% of offspring in each group [39]. Most females give birth in each breeding attempt, usually on the same day [40], and the communal litter is reared by the whole group [41,42]. Pups compete for food and access to helpers, and the *per capita* reproductive success of females declines as the number of breeding females grows large [15]. There is also conspicuous helping behaviour exhibited by both parents and non-parents. Both males and females ‘babysit’ offspring at the den in the first month after birth [41], and after pups emerge they are guarded and provisioned by adult ‘escorts' [43].

In this paper, we investigated what triggers eviction events in groups of banded mongooses. We tested three distinct but non-exclusive hypotheses: (i) eviction is a response to reproductive competition; (ii) eviction is used to coerce cooperation; and (iii) eviction is an adaptive forced dispersal strategy. We make the following predictions (table 1). First, if eviction is a response to reproductive competition we predict that an eviction event is more likely to occur when intrasexual competition is high, and when ecological conditions are unfavourable for successful reproduction. Other things being equal, increasing relatedness should reduce the probability of an eviction event, because dominants should be more tolerant of kin competitors [44], and because kinship should reduce competitive effort within groups [45,46]. Second, if eviction is used to coerce helpers we predict a higher probability of eviction following breeding attempts where helping performance was poor, where the outside options for helpers are good [47,48] and where relatedness is low [49]. In addition, if eviction is used as a mechanism to enforce harder work, we expect eviction events to result in improved helping performance in the subsequent breeding attempt. Third, if eviction is a means by which dominants force copies of their alleles into the wider population we expect eviction events to occur when relatedness in the group is high, when local competition is high, and when ecological conditions are favourable for successful dispersal.

**Table 1.** Predicted effects of social and environmental variables on the probability of eviction under the three hypotheses described in the text. (Numbered references provide theoretical or empirical support for the predictions.)

hypothesis	number of competitors	quality of ecological conditions	prior helping performance^a^	change in helping performance^a^ following eviction	mean group relatedness
reproductive competition	more same-sex competitors 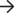 more intrasexual competition 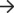 more evictions	poorer conditions 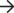 more intrasexual competition 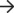 more evictions	no clear prediction	no clear prediction	lower relatedness 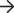 more intrasexual competition [45,46] 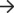 more evictions
coercion of cooperation	no clear prediction	better conditions 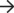 groups less stable [48], or helpers work less hard [47] 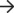 more evictions	poorer helping performance 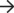 more evictions	positive change 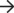 more evictions	lower relatedness 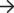 groups less stable [48], or more coercion required [49] 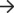 more evictions
adaptive forced dispersal	larger group size 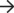 more resource competition 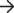 more evictions, or more same-sex competitors 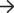 more reproductive competition 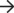 more evictions	better conditions 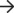 more successful dispersal 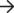 more evictions	no clear prediction	no clear prediction	higher relatedness 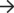 forced dispersal more effective 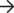 more evictions

^a^Measured by outcome or helping effort.

We tested these predictions using a dataset of 496 breeding attempts for which we had information on group composition, reproductive success, helping behaviour, relatedness, ecological conditions and whether eviction occurred. Note in this paper we explicitly focus on the factors that trigger group eviction events, rather than on what features of individuals determine the risk of being evicted.

## 2. Material and methods

### (a) Study population and data collection

We studied a population of banded mongooses on the Mweya Peninsula, Queen Elizabeth National Park, Uganda (0°12′ S, 27°54′ E), between October 1996 and February 2013. Details of habitat are given elsewhere [38]. Daily measurements of temperature and rainfall were recorded by the Uganda Institute of Ecology Meteorological Station and, later, using our own weather station. Over the 16-year study period, we observed 496 breeding attempts in 16 groups. Following [40], we defined a communal litter as one where all pregnant females gave birth within 30 days of one another. We defined a breeding attempt as the 67 day period prior to the birth of each litter (comprised a 7 day oestrus and a 60 day gestation [50]). We defined an eviction event to have occurred in a breeding attempt if one or more individuals left their group for at least 1 day following a period of intense aggression towards themselves or other group members [15,36]. In practice, evictions are conspicuous and noisy events that are easy to recognize. Typically, individuals leave only after being repeatedly attacked, but much aggression occurs in the bushes where we are unable to identify the aggressors or their victims. Instances where individuals left their group without any observed aggression towards any group member were defined as voluntary dispersal events and were not considered in our analysis. Groups were visited every 1–3 days to record life-history and behavioural data. Most were habituated to human presence, allowing observers to watch and follow them from less than 5 m. One or two individuals in each group wore a radio collar (Sirtrack Ltd., Havelock North, New Zealand) with a 20 cm whip antenna (Biotrack Ltd., UK) that enabled groups to be located. Individuals were easily identifiable by either colour-coded plastic collars or, more recently, unique shave markings on their back. Individuals were regularly trapped to maintain these identification markings (see [51] for details). On first capture, a 2 mm skin sample was collected from the end of the tail using sterilized scissors for genetic analyses. DNA was extracted and used to assign parentage and estimate relatedness using a panel of 43 polymorphic microsatellite markers (see [52] for further details).

### (b) Statistical analyses

We used an information-theoretic approach [53] in which we compared the explanatory power of models to investigate the factors that predict the probability that:

(i) an eviction event occurred in a breeding attempt (‘female evictions’). Since females are evicted in every eviction event, we focused the analysis on the factors predicted to influence female eviction;
(ii) when an eviction event occurred, males were evicted alongside females (‘male evictions’). Here we focused the analysis on the factors predicted to influence male eviction; and
(iii) when an eviction event occurred, it was temporary rather than permanent (‘temporary evictions’). Since temporary evictions could be either female only or mixed-sex events, we included factors predicted to influence both male and female eviction. An eviction was defined as temporary if more than 50% of the evicted cohort were allowed to return to their group.

For each analysis, we constructed a candidate set of models which together provided a comprehensive test of the predictions of our three hypotheses: reproductive competition, coercion of cooperation and adaptive forced dispersal. The models incorporated additive combinations of the main terms predicted to influence eviction probability for the hypotheses, together with specific two-way interactions where we considered these to be biologically relevant.

#### (i) Models of eviction as a response to reproductive competition

To test whether an eviction event is more likely to occur when reproductive competition is high, we fitted the number of reproductive competitors at the start of the breeding attempt (denoted *B*), mean monthly rainfall (mm) (*E*) in the previous six months, the interaction between these social and ecological variables (*B* : *E*), and mean group relatedness (*R*) as fixed effects. Rainfall and insect abundance are correlated ([38,54], H. H. Marshall 2013–2016, unpublished data) so we expect low rainfall to intensify competition for food resources. In the female evictions analysis, reproductive competitors were defined as females 10 months and over (10 months is the age at first conception; [15,55]). In the male evictions analysis, reproductive competitors were defined as males 3 years and over (3 years is the first age at which males typically become regular mate guards; [37]). In the temporary evictions analysis, male and female reproductive competitors were defined as above and fitted as separate fixed effects.

#### (ii) Models of eviction as coerced cooperation

The coercion of cooperation hypothesis predicts that eviction should be triggered by poor helper performance, but it is not clear whether animals should respond to the outcome of helping (i.e. reproductive success), or to helping behaviour *per se*. We separately investigated these alternatives by using two indices of helping performance: (i) female reproductive success (*C*_S_) and (ii) helping effort (*C*_E_). We also examined the change in helping performance (Δ*C*_S_ or Δ*C*_E_).

(i) Female reproductive success, *C*_S_, was defined as the number of emergent pups in the previous breeding attempt, per female that contributed to the communal litter. To account for differences in *C*_S_ that could be explained by differences in the amount of help available, we included the number of helpers available to babysit that litter (*H*) and the interaction between these terms (*C*_S_ : *H*)*.* The interaction term is necessary to capture the difference between the same reproductive outcome achieved with few helpers versus many helpers. We included mean group relatedness (*R*) and mean monthly rainfall (*E*) as main effects. In the female evictions analysis, we defined helpers as females aged six months to 3 years, since females younger than 3 years are classed as subordinate and are more likely to participate in helping [43,56]. In the male evictions analysis, helpers were defined as males aged six months to 3 years, since males do not become consistent breeders until around 3 years of age and, until then, contribute more to helping [37,57]. In the temporary evictions analysis, male and female helpers were defined as above and fitted as separate fixed effects.

To investigate whether eviction is used to coerce helpers to work harder in the subsequent breeding attempt, we tested whether the change in helping performance from one litter to the next predicted the probability that an eviction event occurred in the interim. We reasoned that if eviction is used as a punishment to improve future helping performance, an eviction event (and temporary eviction events in particular) should be associated with an increase in helping performance of remaining or returning helpers after eviction. We fitted Δ*C*_S_, Δ*H* and the interaction between them (Δ*C*_S_ : Δ*H*) as fixed effects, where Δ*C*_S_ is the change in female reproductive success (i.e. the number of emergent pups, per female that contributed to the litter), and Δ*H* is the change in the number of available helpers, across two consecutive breeding attempts (the breeding attempt before the eviction, and the subsequent breeding attempt). Again, we included mean group relatedness (*R*) and mean monthly rainfall (*E*) as fixed effects. Note that Δ*C*_S_ and Δ*H* are likely to be affected by the problem of regression to the mean [58] because extremely high or low values in the first measure of a given variable are more likely to move closer to the mean in a second measure of that variable. We controlled for potential problems with regression to the mean following the methods in [58] (see the electronic supplementary material).

(ii) Helping effort, *C*_E_, was defined as the contribution by helpers (*H*) to babysitting in the previous breeding attempt (i.e. *C*_E_ = number of helpers that babysat per day of babysitting). We repeated the analyses outlined above, replacing *C*_S_ with *C*_E_*.* In the female evictions analysis, *C*_E_ was defined as the number of female babysitters aged six months to 3 years left per day of babysitting of the previous litter. In the male evictions analysis, *C*_E_ was defined number of male babysitters aged six months to 3 years left per day of babysitting of the previous litter. In the temporary evictions analysis, *C*_E_ was defined as in the previous two analyses and fitted as separate fixed effects. In the temporary eviction analysis, the model including both the change in female helpers' babysitting effort and male helpers' babysitting effort was too complex to fit to the reduced sample of data and so these variables were fitted in separate models. Since data on babysitting behaviour was not available for all breeding attempts, analysis using this helping effort measure of helping performance was performed on a reduced sample (see the electronic supplementary material, tables S2, S4 and S6).

#### (iii) Models of eviction as an adaptive forced dispersal strategy

To test whether an eviction event is more likely to occur when relatedness is high, ecological conditions are good and local competition is intense, we fitted mean group relatedness (*R*), mean monthly rainfall (*E*), group size (all individuals aged over six months) (*G*), the interaction between relatedness and rainfall (*R* : *E*), and the interaction between relatedness and group size (*R* : *G*) as fixed effects. We included group size to allow for the possibility that local resource competition contributes to the timing of eviction events. The interaction between relatedness and rainfall is particularly important to test the prediction that high group relatedness in combination with favourable ecological conditions will make an eviction event more likely to occur. The definitions of *R*, *E* and *G* were consistent across our three analyses. An alternative prediction is that the nature of competition under which adaptive forced dispersal operates could be reproductive, rather than resource related. We fitted an identical set of models to those described above, but replaced *G* with the number of reproductive competitors (*B*) in each of the three analyses.

#### (iv) Comparing model performance

Models, including a null model containing no fixed effects, were estimated using generalized linear mixed models (GLMM). Group identification (ID) was included as a random intercept to control for repeated measures across groups. In all analyses, we used the maximum sample size for which we had data on all the terms in all the models (electronic supplementary material, tables S1–S6). In all three analyses, the eviction metric was fitted as the binomial response variable using a logit link function in the lme4 package in R v. 3.1.2 [59,60]. We performed subsets selection of the maximal model under each hypothesis using the ‘MuMIn’ package [61], which examines all possible combinations of terms in each full model. Models were ranked by Akaike's information criteria (AIC), or corrected AIC (AICc) in analyses where *N/k* < 40, where *N* is the sample size and *k* is the number of parameters in the maximal model [53]. We defined a ‘top model set’ as models ≤ Δ6 AIC (or AICc) units of the best supported model [62], after excluding any models where a simpler nested version attained stronger support (applying the ‘nesting rule’ of [62]). Full model tables are provided in the electronic supplementary material.

## 3. Results

### (a) Observations of eviction

In total, we observed 47 eviction events in eight out of 16 groups in our population between October 1996 and February 2013 resulting in the expulsion of 457 individuals. More females than males were evicted; in the 46 events for which we knew the sex and identities of the evictees, evictions resulted in the expulsion of 274 females and 170 males, with the median evicted cohort comprising 24% of the total group (range 3%–60%). Just three eviction events (6%) resulted in the eviction of a single individual. In 25 (53%) of eviction events only females were evicted, with a median of six females evicted in a single event (range 1–12). On average, an eviction event resulted in the expulsion of 40% of female group members (range 6–79%). In the remaining 22 eviction events (47%), a cohort of males was evicted alongside a cohort of females. In these cases, the median number of evictees was 13 individuals (range 6–26); median number of female evictees was six (range 2–15) and median number of male evictees was nine (range 1–17). On average, an eviction event resulted in the expulsion of 35% of male group members (range 3–65%). Males were only ever evicted alongside females. In eight out of 22 mixed-sex evictions (36%), some or all of both sexes dispersed permanently as a consequence of eviction. In all these cases, the evicted cohorts of males and females split into single-sex groups and dispersed separately. In 47% of all eviction events, all evictees were eventually readmitted to their group after persistently attempting to re-join. In 32%, some evicted individuals (both males and females) were allowed to return but others were not. Of temporarily evicted individuals, 69% were readmitted to their group within one week, 97% within one month and all individuals within six months of eviction.

### (b) Testing the hypotheses

#### (i) Female evictions

**Figure 1.**
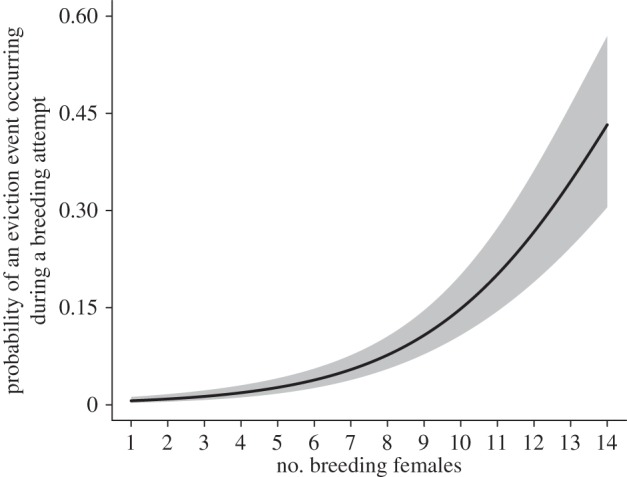
The probability of an eviction event occurring during a breeding attempt against the number of breeding females (*N* = 415 breeding attempts in 15 groups). The line shows model predictions (±s.e.).

Models of the reproductive competition hypothesis were by far the best predictors of the probability of an eviction event occurring during a breeding attempt (table 2). Specifically, it was the model containing the number of breeding females that performed the best out of the candidate model set, with an eviction event more likely to occur when there were more breeding females (figure 1). Models of the reproductive competition hypothesis had a cumulative adjusted Akaike's model weight of 100% of retained models from the top model set when helping performance was measured in terms of female reproductive success (*C*_S_) (table 2), and 95% when helping performance was measured in terms of helping effort (*C*_E_) (electronic supplementary material, table S2).

**Table 2.** Female evictions. (Model performance in predicting the probability of an eviction event occurring during a breeding attempt (*N* = 415 breeding attempts in 15 groups). Analysis using the female reproductive success (*C*_S_) measure of helping performance under the coercion of cooperation hypothesis. Models comprise the top model set where ΔAIC ≤ 6. Hyp., hypothesis; A, adaptive forced dispersal; R, reproductive competition. Columns 2–8 show parameter effect sizes from GLMMs on the logit scale: Int., Intercept; *B,* number of breeding females; *E,* mean rainfall in previous six months; *R,* mean group relatedness; symbol ‘:’, interaction; *k*, number of estimated parameters including a random intercept for group ID; logLik, log-likelihood; AIC, Akaike's information criterion; ΔAIC, change in AIC value from the best performing model; *w_i_*, Akaike's model weight; retained, ticks indicate that the model was retained after applying the nesting rule of [62]; Adj. *w_i_*, adjusted Akaike's model weight for the retained models. Blank cells indicate that the term was absent from that model.)

Hyp.	Int.	*B*	*E*	*R*	*B* : *E*	*R* : *B*	*R* : *E*	*k*	logLik	AIC	ΔAIC	*w_i_*	retained	Adj. *w_i_*
R	−5.44	0.37						3	−108.63	223.26	0.00	0.34	✓	1.00
A	−3.34	0.11		−14.46		1.76		5	−107.25	224.50	1.24	0.18		
A/R	−5.49	0.37		0.42				4	−108.62	225.25	1.99	0.13		
R	−5.45	0.37	0					4	−108.63	225.26	2.00	0.13		
A	−3.29	0.11	0	−14.52		1.77		6	−107.25	226.50	3.24	0.07		
A/R	−5.51	0.37	0	0.43				5	−108.62	227.24	3.99	0.05		
R	−5.37	0.36	0		0			5	−108.63	227.26	4.00	0.05		
A	−3.34	0.11	0	−14.11		1.77	−0.01	7	−107.25	228.49	5.23	0.02		
A	−5.25	0.37	0	−1.44			0.03	6	−108.60	229.21	5.95	0.02		
R	−5.42	0.36	0	0.44	0			6	−108.62	229.24	5.98	0.02		

#### (ii) Male evictions

**Figure 2.**
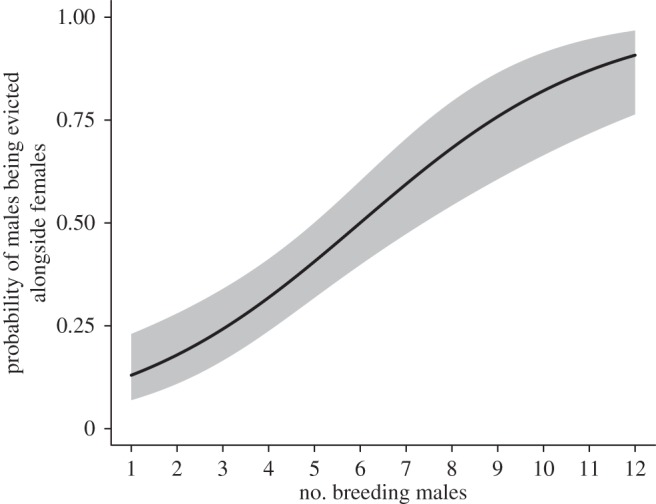
The probability that males are evicted alongside females when an eviction event occurs (*N* = 37 eviction events in seven groups). The line shows model predictions (±s.e.).

The probability that males were evicted with females, given that an eviction occurred, was also best explained by the reproductive competition hypothesis (analysis using the female reproductive success (*C*_S_) measure of helping performance). Specifically, the model that performed best contained the number of breeding males (table 3), with males more likely to be evicted with females as the number of breeding males increased (figure 2). The only other model to be retained after applying the nesting rule [62] was the model of adaptive forced dispersal containing group size and mean group relatedness, with males more likely to be evicted alongside females in larger groups and when group relatedness was low, although this model only attained an adjusted weight of 5%. When performing the same analysis but using the helping effort (*C*_E_) measure of helping performance on a reduced sample size, the only model that was retained was the null model which contained an intercept but no fixed effects (electronic supplementary material, table S4).

**Table 3.** Male evictions. (Model performance in predicting the probability that males are evicted alongside females when an eviction event occurs (*N* = 37 eviction events in seven groups). Analysis using the female reproductive success (*C*_S_) measure of helping performance under the coercion of cooperation hypothesis. Models comprise the top model set where ΔAICc ≤ 6. Hyp., hypothesis; A, adaptive forced dispersal; R, reproductive competition; column headings as in table 2, with the addition of *B*, number of breeding males; *G* = group size; AICc, corrected Akaike's information criterion; ΔAICc, change in AICc value from the best performing model. Ticks indicate that the model was retained after applying the nesting rule of [62]. Blank cells indicate that the term was absent from that model.)

Hyp.	Int.	*B*	*E*	*R*	*B : E*	*R : B*	*G*	*k*	logLik	AICc	ΔAICc	*w_i_*	retained	Adj. *w_i_*
R	−2.28	0.38						3	−20.42	47.57	0.00	0.51	✓	0.95
R	−1.81	0.39	−0.01					4	−20.32	49.88	2.32	0.16		
A/R	−2.16	0.38		−0.68				4	−20.41	50.07	2.51	0.15		
R	−0.30	−0.10	−0.04		0.01			5	−19.78	51.51	3.94	0.07		
A	−0.94	0.11		−9.71		2.02		5	−20.24	52.41	4.85	0.05		
A/R	−1.64	0.39	−0.01	−0.90				5	−20.31	52.55	4.98	0.04		
A	−3.82			−1.58			0.15	4	−22.08	53.41	5.84	0.03	✓	0.05

#### (iii) Temporary evictions

None of our hypotheses explained whether eviction events were temporary rather than permanent. The null model performed better than all other models and this result was consistent whether female reproductive success (*C*_S_) or helping effort (*C*_E_) was used as a measure of helping performance (electronic supplementary material, tables S5 and S6).

## 4. Discussion

Previous work on eviction in this species highlighted reproductive competition as a driver of female evictions, but did not consider male or temporary evictions, or test alternative hypotheses for eviction behaviour [8,15,36]. For both female and mixed-sex eviction events, the reproductive competition hypothesis best explained our data. Females were more likely to be evicted when there were many breeding females in the group. These female eviction events are likely to radically alter the landscape of intrasexual competition among remaining males, which may explain why groups of males are commonly evicted alongside females. Males were more likely to be evicted when there were many breeding males in the group, again supporting the hypothesis that high levels of same-sex reproductive competition is a trigger for mass eviction.

Sex differences in the intensity of reproductive competition may explain why evictions of females are almost twice as common as male evictions. Reproductive competition is particularly intense among female banded mongooses because dominants are unable to suppress reproduction by younger females and suffer substantial fitness costs when large numbers of subordinate females breed alongside them [15,56]. Dominant males, by contrast, can usually prevent subordinate males from mating, and so are less sensitive to the presence of additional males in the group. However, dominant males are not immune from reproductive competition because they cannot fully control the mating behaviour of females [39,50]. Dominant males might also evict (usually younger) subordinates before these become genuine reproductive competitors, similar to the explanations for eviction in size-based fish hierarchies [17–20]. At the same time, young male banded mongooses that are excluded from breeding have less to gain from putting up a fight to stay in their natal group compared with females. This potential difference in the level of resistance offered could explain why males sometimes disperse voluntarily, while female dispersal events almost always involve intense aggression.

We found little evidence to support the idea that mass evictions are triggered when it is adaptive for dominants to force subordinates to disperse. We did find weak support for a model which showed that males were more likely to be evicted with females when groups were large, but when mean group relatedness was low. This effect of relatedness is the opposite of that predicted under the adaptive forced dispersal hypothesis. Eviction of either sex was not more likely when mean group relatedness was high, or when ecological conditions were benign. We cannot rule out adaptive forced dispersal entirely, however, because: (i) we currently lack information about the long-term fate of evictees in the wider population and (ii) we currently lack a formal model of the adaptive forced dispersal hypothesis which might provide discriminating predictions beyond those based on our simple verbal arguments. Concerning point (i), eviction did result in the permanent dispersal of 193 individuals, which is 72% of the individuals in our population that left their natal group [37]. Eviction is therefore likely to be a major determinant of gene flow and population structure in this system. Concerning (ii), demographic models of kin selection [13,63] usually assume that dispersal is under the full control of the offspring themselves, or under full maternal control (e.g. [64], but see [65]). Our observations of eviction, by contrast, suggest that in many real systems, no single party has full control over group membership, and group dynamics are a compromise between the interests of evictors and evictees. A model embedding a conflict resolution mechanism (e.g. similar to Higashi & Yamamura's [44] insider–outsider conflict model) in a demographic framework could be a useful tool to predict population consequences of reproductive competition.

Finally, we found little evidence to support the coercion of cooperation hypothesis for mass eviction in this system. This contrasts with strong evidence that eviction, and the threat of eviction, is used to coerce helpers to work harder in the cooperative cichlid *N. pulcher* [9,24,25,49,66]. Why should eviction be effective to coerce cooperation in cichlids but not banded mongooses? We suggest two reasons. First, theory suggests that acts and threats of eviction will be much less effective at coercing cooperation when targeted at a group of individuals rather than specific individual helpers [15]. In a group of helpers, the threat of mass eviction creates a tragedy-of-the-commons over helping effort since the effort of any hard working helper can be readily exploited by the idleness of other potential evictees. Eviction is likely to be much more effective at inducing cooperation when targeted at individual transgressors; for example, in dyads and in groups which exhibit a strict rank hierarchy (such as cooperative cichlids; [9,19,49]). Second, threats of eviction are predicted to be less effective at inducing pre-emptive cooperation when evictees are often reaccepted into the group, as in banded mongooses ([15]; this paper) and meerkats [16]. The best tests of the coercion of cooperation hypothesis require experimental reduction of helper effort [9,24], or manipulation of the availability of outside options [66,67], which is logistically challenging in birds and mammals. Further innovative experimental tests in a wider range of cooperative vertebrates would help to test the coercion of cooperation hypothesis more rigorously.

To summarize, our results suggest that intrasexual reproductive competition is the trigger for mass eviction of both sexes from groups of banded mongooses. Eviction of females appears to alter the landscape of intrasexual competition among males, leading to the mass eviction of males at the same time as, but separate from, the eviction of females. We did not find evidence to link eviction events to the enforcement of helping or the propagation of alleles through a structured population. Nevertheless, our study highlights that the consequences of resolving within-group reproductive competition can scale up to affect population structure and demography. This link between within-group conflict strategies and population processes has been little studied theoretically or empirically, but may be an important determinant of life-history evolution in viscous animal societies.

## Supplementary Material

Thompson et al Electronic Supplementary Material

## Data accessibility

Data is available from the Dryad Digital Repository: http://dx.doi.org/10.5061/dryad.8c26b.
