# Supplementary material for: Reproductive competition triggers mass eviction in cooperative banded mongooses
Source: Proc Biol Sci. 2016 Mar 16;283(1826):20152607. doi: 10.1098/rspb.2015.2607 (PMC4810850; doi:10.1098/rspb.2015.2607)
Supplement: Thompson et al Electronic Supplementary Material [file rspb20152607supp1.pdf]

Electronic Supplementary Material

Controlling for regression to the mean

To account for potential problems with regression to the mean, we calculated ΔC_S, ΔC_E and ΔH as an adjusted change using the formulae from Kelly & Price (2005):

$$\Delta X = (X_2 - \bar{X}_2) - \hat{p}(X_1 - \bar{X}_1)$$

and

$$\hat{p} = \frac{2rs_1s_2}{{s_1}^2 + {s_2}^2}$$

where X_1 and s_1 are the observation and standard deviation of the observation in the breeding attempt before eviction, and X_2 and s_2 are the observation and standard deviation of the observation in the breeding attempt of the eviction, and r is the correlation between the observations in the two consecutive breeding attempts.

ESM Table 1: ‘Female evictions’. Model performance in predicting the probability of an eviction event occurring during a breeding attempt (N=415 breeding attempts in 15 groups). Analysis using the female reproductive success (C_S) measure of helping performance under the coercion of cooperation hypothesis. Models above the line comprise the top model set where ΔAIC ≤ 6.

**Hyp.**	**Int.**	C_S	H	$C_S:H$	R	E	ΔC_S	ΔH	$\Delta C_S:\Delta H$	B	$B:E$	$R:E$	G	$R:G$	$R:B$	**k**	**logLik**	**AIC**	**ΔAIC**	w_i	**Retained**	**Adj.** w_i
R	-5.44									0.37						3	-108.63	223.26	0.00	0.34	✓	1.00
A	-3.34				-14.46					0.11					1.76	5	-107.25	224.50	1.24	0.18		
A/R	-5.49				0.42					0.37						4	-108.62	225.25	1.99	0.13		
R	-5.45					0.00				0.37						4	-108.63	225.26	2.00	0.13		
A	-3.29				-14.52	0.00				0.11					1.77	6	-107.25	226.50	3.24	0.07		
A/R	-5.51				0.43	0.00				0.37						5	-108.62	227.24	3.99	0.05		
R	-5.37					0.00				0.36	0.00					5	-108.63	227.26	4.00	0.05		
A	-3.34				-14.11	0.00				0.11		-0.01			1.77	7	-107.25	228.49	5.23	0.02		
A	-5.25				-1.44	0.00				0.37		0.03				6	-108.60	229.21	5.95	0.02		
R	-5.42				0.44	0.00				0.36	0.00					6	-108.62	229.24	5.98	0.02		
C	-4.99	0.47	0.40	-0.07												5	-111.65	233.30	10.04	0.00		
C	-5.07	0.47	0.40	-0.07	0.57											6	-111.63	235.27	12.01	0.00		
C	-4.97	0.47	0.40	-0.07		0.00										6	-111.65	235.30	12.04	0.00		
C	-5.05	0.46	0.40	-0.07	0.57	0.00										7	-111.63	237.27	14.01	0.00		
A	-2.75				-11.13								0.01	0.56		5	-115.16	240.31	17.05	0.00		
A	-4.32				1.65								0.07			4	-116.75	241.50	18.24	0.00		
A	-2.98				-10.94	0.00							0.01	0.56		6	-115.10	242.21	18.95	0.00		
A	-4.60				1.76	0.00							0.07			5	-116.66	243.33	20.07	0.00		
A	-2.65				-13.35	0.00						0.04	0.01	0.56		7	-115.07	244.14	20.88	0.00		
A	-4.19				-1.11	0.00						0.05	0.07			6	-116.62	245.25	21.99	0.00		
C	-2.86						0.37	0.11	0.01							5	-118.19	246.37	23.11	0.00		
Null	-2.87															2	-121.28	246.57	23.31	0.00		
C	-3.37					0.01	0.38	0.11	0.01							6	-117.86	247.71	24.45	0.00		
C	-3.19				2.03		0.38	0.11	0.01							6	-117.98	247.96	24.70	0.00		
Any	-3.15				1.69											3	-121.14	248.28	25.02	0.00		
C	-3.80				2.32	0.01	0.39	0.11	0.01							7	-117.59	249.19	25.93	0.00		
Any	-3.62				1.93	0.01										4	-120.90	249.81	26.55	0.00		
A	-3.00				-2.86	0.00						0.08				5	-120.79	251.57	28.31	0.00		

Hyp. = Hypothesis: A = Adaptive Forced Dispersal; C = Coercion of Cooperation; R = Reproductive Competition; Any = Any of the three hypotheses; Null = null model. Columns 2 to 16 show parameter effect sizes from GLMMs on the logit scale: Int. = Intercept; C_S = number of emergent pups per female that contributed to the previous litter; H = number of female helpers in the

previous breeding attempt; R = mean group relatedness; E = mean rainfall in previous 6 months; ΔC_S = change in the number of emergent pups per female that contributed to the litter in the breeding attempts before and of the eviction; ΔH = change in the number of female helpers in the breeding attempts before and of the eviction; B = number of breeding females; G = group size; : = interaction. k = number of estimated parameters including a random intercept for group ID; logLik = log-likelihood; AIC = Akaike's information criterion; ΔAIC = change in AIC value from the best performing model; w_i = Akaike's model weight; Retained = tick indicates that the model was retained after applying the nesting rule of Richards et al. (2011); Adj. w_i = adjusted Akaike's model weight for the retained models. Blank cells indicate that the term was absent from that model.

ESM Table 2: ‘Female evictions’. Model performance in predicting the probability of an eviction event occurring during a breeding attempt (*N*=270 breeding attempts in 14 groups). Analysis using the helping effort (C_E) measure of helping performance under the coercion of cooperation hypothesis. Models above the line comprise the top model set where ΔAICc ≤ 6.

**Hyp.**	**Int.**	C_E	H	C_E:H	R	E	ΔC_E	ΔH	ΔC_E:ΔH	B	B:E	R:E	G	R:G	R:B	**k**	**logLik**	**AICc**	**ΔAICc**	w_i	**Retained**	**Adj.** w_i
R	1.28					-0.11				-0.67	0.02					5	-62.26	134.76	0.00	0.27	✓	0.49
R	1.66				-3.95	-0.11				-0.62	0.02					6	-61.82	135.96	1.20	0.15		
R	-7.65					0.03				0.43						4	-64.04	136.22	1.47	0.13	✓	0.24
R	-5.73									0.44						3	-65.16	136.41	1.65	0.12	✓	0.22
A/R	-6.95				-4.25	0.03				0.45						5	-63.50	137.23	2.48	0.08		
A/R	-5.10				-4.46					0.46						4	-64.55	137.24	2.49	0.08		
A	-6.67				4.71					0.66					-1.13	5	-64.36	138.95	4.20	0.03		
A	-8.49				4.67	0.03				0.64					-1.10	6	-63.33	138.99	4.23	0.03		
A	-8.18				3.23	0.05				0.45		-0.12				6	-63.43	139.17	4.42	0.03		
C	-5.30	-5.16	0.17	0.79		0.03										6	-64.13	140.58	5.83	0.01	✓	0.03
C	-3.44	-5.13	0.18	0.79												5	-65.18	140.60	5.84	0.01	✓	0.02
A	-9.65				11.65	0.05				0.64		-0.11			-1.07	7	-63.27	140.96	6.21	0.01		
C	-3.02	-5.04	0.18	0.79	-2.66											6	-64.95	142.21	7.46	0.01		
C	-4.86	-5.11	0.17	0.79	-2.45	0.03										7	-63.94	142.30	7.55	0.01		
A	-4.97				-1.57								0.13			4	-67.35	142.85	8.10	0.00		
A	-6.56				-1.51	0.03							0.13			5	-66.51	143.24	8.48	0.00		
A	-4.45				-4.97								0.11	0.15		5	-67.33	144.88	10.12	0.00		
A	-7.75				5.59	0.05						-0.11	0.12			6	-66.44	145.20	10.44	0.00		
A	-5.84				-6.40	0.03							0.09	0.22		6	-66.45	145.22	10.47	0.00		
A	-7.02				0.63	0.05						-0.12	0.09	0.24		7	-66.37	147.18	12.42	0.00		
Null	-2.61															2	-75.89	155.83	21.07	0.00		
Any	-5.13				-0.03	0.04										4	-74.03	156.21	21.46	0.00		
C	-5.09					0.04	0.26	-0.54	0.19							6	-72.56	157.45	22.69	0.00		
Any	-2.63				0.15											3	-75.89	157.87	23.12	0.00		
A	-6.61				9.04	0.06						-0.14				5	-73.91	158.05	23.30	0.00		
C	-2.58						0.25	-0.49	0.11							5	-74.37	158.98	24.22	0.00		
C	-5.04				-0.23	0.04	0.26	-0.53	0.19							7	-72.56	159.55	24.80	0.00		
C	-2.53				-0.24		0.25	-0.49	0.11							6	-74.37	161.07	26.31	0.00		

Hyp. = Hypothesis: A = Adaptive Forced Dispersal; C = Coercion of Cooperation; R = Reproductive Competition; Any = Any of the three hypotheses; Null = null model. Columns 2 to 16 show parameter effect sizes from GLMMs on the logit scale: Int. = Intercept; C_E = number of female helpers left per day of babysitting of the previous litter; *H* = number of female helpers in the

previous breeding attempt; R = mean group relatedness; E = mean rainfall in previous 6 months; ΔC_E = change in the number of female helpers left per day of babysitting in the breeding attempts before and of the eviction; ΔH = change in the number of female helpers in the breeding attempts before and of the eviction; B = number of breeding females; G = group size; : = interaction. k = number of estimated parameters including a random intercept for group ID; logLik = log-likelihood; AICc = corrected Akaike's information criterion; ΔAICc = change in AICc value from the best performing model; w_i = Akaike's model weight; Retained = tick indicates that the model was retained after applying the nesting rule of Richards et al. (2011); Adj. w_i = adjusted Akaike's model weight for the retained models. Blank cells indicate that the term was absent from that model.

ESM Table 3: ‘Male evictions’. Model performance in predicting the probability that males are evicted alongside females when an eviction event occurs (N=37 eviction events in 7 groups). Analysis using the female reproductive success (C_S) measure of helping performance under the coercion of cooperation hypothesis. Models above the line comprise the top model set where ΔAICc ≤ 6.

Hyp.	Int.	C_S	H	C_S:H	R	E	ΔC_S	ΔH	ΔC_S:ΔH	B	B:E	R:E	G	R:G	R:B	k	logLik	AICc	ΔAICc	w_i	Retained	Adj. w_i
R	-2.28									0.38						3	-20.42	47.57	0.00	0.47	✓	0.95
R	-1.81					-0.01				0.39						4	-20.32	49.88	2.32	0.15		
A/R	-2.16				-0.68					0.38						4	-20.41	50.07	2.51	0.13		
R	-0.30					-0.04				-0.10	0.01					5	-19.78	51.51	3.94	0.07		
A	-0.94				-9.71					0.11					2.02	5	-20.24	52.41	4.85	0.04		
A/R	-1.64				-0.90	-0.01				0.39						5	-20.31	52.55	4.98	0.04		
A	-3.82				-1.58								0.15			4	-22.08	53.41	5.84	0.03	✓	0.05
Null	-0.38															2	-24.98	54.31	6.75	0.02		
R	-0.25				-0.35	-0.04				-0.10	0.01					6	-19.78	54.37	6.80	0.02		
A	-0.21				-10.96	-0.01				0.09					2.25	6	-20.10	55.00	7.44	0.01		
A	-1.35				-3.19	-0.01				0.39		0.03				6	-20.30	55.40	7.84	0.01		
A	-3.45				-1.54	-0.01							0.16			5	-21.90	55.74	8.18	0.01		
A	-6.12				14.14								0.25	-0.70		5	-21.95	55.84	8.27	0.01		
Any	0.09				-2.97											3	-24.83	56.38	8.82	0.01		
A	0.11				-13.50	-0.01				0.08		0.04			2.26	7	-20.10	58.05	10.49	0.00		
A	-5.84				14.56	-0.01							0.26	-0.71		6	-21.78	58.36	10.80	0.00		
A	-2.14				-11.17	-0.03						0.14	0.16			6	-21.85	58.49	10.93	0.00		
Any	0.18				-3.00	0.00										4	-24.82	58.90	11.33	0.00		
C	-1.61	0.38	0.11	-0.02												5	-24.15	60.24	12.68	0.00		
C	-0.53						0.35	0.01	-0.05							5	-24.21	60.35	12.78	0.00		
A	2.51				-22.06	-0.03						0.28				5	-24.48	60.90	13.34	0.00		
A	-4.48				4.69	-0.03						0.16	0.27	-0.76		7	-21.71	61.28	13.72	0.00		
C	-1.25	0.37	0.12	-0.02	-2.29											6	-24.07	62.95	15.38	0.00		
C	-1.47	0.38	0.11	-0.02		0.00										6	-24.14	63.09	15.52	0.00		
C	-0.25				-1.74		0.33	0.02	-0.05							6	-24.16	63.12	15.55	0.00		
C	-0.81					0.00	0.37	0.01	-0.05							6	-24.17	63.14	15.57	0.00		
C	-1.10	0.37	0.12	-0.02	-2.33	0.00										7	-24.06	65.98	18.42	0.00		
C	-0.52				-1.55	0.00	0.35	0.01	-0.05							7	-24.13	66.12	18.56	0.00		

Hyp. = Hypothesis: A = Adaptive Forced Dispersal; C = Coercion of Cooperation; R = Reproductive Competition; Any = Any of the three hypotheses; Null = null model. Columns 2 to 16 show parameter effect sizes from GLMMs on the logit scale: Int. = Intercept; C_S = number of emergent pups per female that contributed to the previous litter; H = number of male helpers in the

previous breeding attempt; *R* = mean group relatedness; *E* = mean rainfall in previous 6 months; ΔC_S = change in the number of emergent pups per female that contributed to the litter in the breeding attempts before and of the eviction; Δ*H* = change in the number of male helpers in the breeding attempts before and of the eviction; *B* = number of breeding males; *G* = group size; : = interaction. k = number of estimated parameters including a random intercept for group ID; logLik = log-likelihood; AICc = corrected Akaike's information criterion; ΔAICc = change in AICc value from the best performing model; w_i = Akaike's model weight; Retained = tick indicates that the model was retained after applying the nesting rule of Richards et al. (2011); Adj. w_i = adjusted Akaike's model weight for the retained models. Blank cells indicate that the term was absent from that model.

ESM Table 4: 'Male evictions'. Model performance in predicting the probability that males are evicted alongside females when an eviction event occurs (N=22 eviction events in 6 groups). Analysis using the helping effort (C_E) measure of helping performance under the coercion of cooperation hypothesis. Models above the line comprise the top model set where ΔAICc ≤ 6.

Hyp.	Int.	C_E	H	C_E:H	R	E	ΔC_E	ΔH	ΔC_E:ΔH	B	B:E	R:E	G	R:G	R:B	k	logLik	AICc	ΔAICc	w_i	Retained	Adj. w_i
Null	0.18															2	-15.16	34.95	0.00	0.31	✓	1.00
R	-1.00									0.24						3	-14.24	35.80	0.86	0.20		
A	-3.25				1.65								0.13			4	-13.56	37.47	2.52	0.09		
Any	0.06				0.70											3	-15.15	37.64	2.69	0.08		
R	-2.36					0.02				0.22						4	-14.07	38.50	3.55	0.05		
R	8.86					-0.16				-2.33	0.04					5	-12.42	38.59	3.65	0.05		
A/R	-1.28				1.53					0.24						4	-14.21	38.77	3.82	0.05		
C	-0.07						-0.11	0.52	0.53							5	-12.83	39.41	4.46	0.03		
Any	-1.98				1.23	0.03										4	-14.83	40.01	5.06	0.02		
A	-1.86				-8.18								0.06	0.46		5	-13.50	40.75	5.81	0.02		
A	-2.66				1.49	-0.02							0.14			5	-13.50	40.76	5.81	0.02		
C	-2.04	0.46	0.27	-0.07												5	-13.92	41.59	6.65	0.01		
A/R	-2.75				1.86	0.02				0.23						5	-14.03	41.82	6.87	0.01		
A	-0.63				-2.63					0.08					1.02	5	-14.18	42.11	7.16	0.01		
R	8.82				3.88	-0.17				-2.40	0.04					6	-12.28	42.16	7.21	0.01		
A	-7.27				36.23	0.11						-0.55				5	-14.51	42.77	7.82	0.01		
C	1.11					-0.02	-0.21	0.58	0.66							6	-12.77	43.13	8.18	0.01		
C	-0.35				1.53		-0.10	0.52	0.53							6	-12.81	43.21	8.27	0.01		
A	-6.95				31.77	0.05						-0.47	0.15			6	-13.27	44.14	9.19	0.00		
A	-1.68				-6.27	-0.01							0.09	0.36		6	-13.47	44.55	9.60	0.00		
A	-7.38				32.88	0.10				0.22		-0.48				6	-13.79	45.18	10.23	0.00		
C	-2.74	0.46	0.25	-0.07		0.01										6	-13.87	45.34	10.40	0.00		
C	-1.87	0.50	0.28	-0.08	-1.25											6	-13.91	45.41	10.47	0.00		
A	-1.84				-5.55	0.03				-0.06					1.83	6	-13.96	45.51	10.56	0.00		
C	0.83				1.48	-0.02	-0.20	0.58	0.66							7	-12.74	47.49	12.54	0.00		
A	-7.32				33.14	0.13						-0.98	-0.08	1.48		7	-12.93	47.85	12.91	0.00		
A	-7.20				30.57	0.12				-0.26		-0.63			2.97	7	-13.59	49.18	14.23	0.00		
C	-2.58	0.49	0.26	-0.08	-1.02	0.01										7	-13.86	49.72	14.78	0.00		

Hyp. = Hypothesis: A = Adaptive Forced Dispersal; C = Coercion of Cooperation; R = Reproductive Competition; Any = Any of the three hypotheses; Null = null model. Columns 2 to 16 show parameter effect sizes from GLMMs on the logit scale: Int. = Intercept; C_E = number of male helpers left per day of babysitting of the previous litter; H = number of male helpers in the

previous breeding attempt; *R* = mean group relatedness; *E* = mean rainfall in previous 6 months; ΔC_E = change in the number of male helpers left per day of babysitting in the breeding attempts before and of the eviction; ΔH = change in the number of male helpers in the breeding attempts before and of the eviction; *B* = number of breeding males; *G* = group size; : = interaction. k = number of estimated parameters including a random intercept for group ID; logLik = log-likelihood; AICc = corrected Akaike's information criterion; ΔAICc = change in AICc value from the best performing model; w_i = Akaike's model weight; Retained = tick indicates that the model was retained after applying the nesting rule of Richards et al. (2011); Adj. w_i = adjusted Akaike's model weight for the retained models. Blank cells indicate that the term was absent from that model.

ESM Table 5: ‘Temporary evictions’. Model performance in predicting the probability that evictees are allowed to return to their group following an eviction event (N=37 eviction events in 7 groups). Analysis using the female reproductive success (C_S) measure of helping performance under the coercion of cooperation hypothesis. Models above the line comprise the top model set where ΔAICc ≤ 6.

Hyp.	Int.	C_S	H_F	H_M	C_S:H_F	C_S:H_M	R	E	ΔC_S	ΔH_F	ΔH_M	ΔC_S: ΔH_F	ΔC_S: ΔH_M	B_F	B_M	B_F:E	B_M:E	R:E	G	R:G	R:B_F	R: B_M	k	logLik	AICc	ΔAICc	w_i	Ret.	Adj. w_i
Null	0.6																						2	-24.0	52.3	0.0	0.2	✓	1.00
R	1.9													-0.1									3	-23.5	53.8	1.5	0.1		
Any	1.4						-4.9																3	-23.6	53.9	1.6	0.1		
R	0.8														0.0								3	-24.0	54.6	2.3	0.1		
C	0.7								0.1	-0.1		-0.5											5	-21.5	54.9	2.6	0.1		
A/R	2.5						-4.5							-0.1									4	-23.2	55.7	3.3	0.0		
Any	2.3						-5.3	0.0															4	-23.3	55.8	3.5	0.0		
R	2.5							0.0						-0.1									4	-23.3	55.9	3.6	0.0		
R	1.9													-0.1	0.0								4	-23.5	56.3	4.0	0.0		
A/R	1.7						-5.2								0.0								4	-23.5	56.3	4.0	0.0		
A	1.3						-4.9												0.0				4	-23.6	56.4	4.1	0.0		
R	1.5							0.0							0.0								4	-23.7	56.6	4.3	0.0		
C	1.7						-5.8		0.1	-0.1		-0.5											6	-21.0	56.7	4.4	0.0		
C	0.7								0.1		0.3		-0.2										5	-22.7	57.4	5.1	0.0		
A	-0.4						13.3							0.2							-2.2		5	-22.8	57.5	5.1	0.0		
C	0.9							0.0	0.1	-0.1		-0.5											6	-21.5	57.7	5.4	0.0		
A	-0.2						15.4	0.0										-0.3					5	-22.9	57.8	5.5	0.0		
A/R	3.2						-4.7	0.0						-0.1									5	-23.0	57.9	5.5	0.0		
C	-0.8	0.8		0.2		-0.1																	5	-23.1	58.2	5.9	0.0		
R	-0.3							0.0						0.2		0.0							5	-23.1	58.2	5.9	0.0		
A/R	2.6						-4.6							-0.1	0.0								5	-23.2	58.3	6.0	0.0		
A	2.0						-5.1	0.0											0.0				5	-23.2	58.4	6.1	0.0		
A/R	2.5						-5.5	0.0							0.0								5	-23.2	58.4	6.1	0.0		
R	2.5							0.0						-0.1	0.0								5	-23.3	58.6	6.3	0.0		
C	0.8								0.0	-0.3	0.3	-0.4	-0.2										7	-20.4	58.7	6.4	0.0		
A	0.8						0.8								0.1							-1.4	5	-23.4	58.7	6.4	0.0		
R	0.5							0.0							0.3		0.0						5	-23.4	58.8	6.5	0.0		
A	1.8						-8.7												0.0	0.2			5	-23.6	59.1	6.7	0.0		
C	1.4	-0.5	-0.1		0.1																		5	-23.6	59.1	6.7	0.0		
C	1.8						-6.4		0.0		0.3		-0.2										6	-22.2	59.2	6.9	0.0		
C	1.6							0.0	0.0		0.3		-0.2										6	-22.4	59.6	7.3	0.0		
C	1.9						-5.9	0.0	0.0	-0.1		-0.5											7	-21.0	59.8	7.5	0.0		
C	0.1	0.8		0.2		-0.1	-5.7																6	-22.6	60.0	7.7	0.0		
A	0.4						11.0	0.0						0.2							-1.9		6	-22.6	60.1	7.8	0.0		

Hyp.	Int.	C_S	H_F	H_M	C_S:H_F	C_S:H_M	R	E	ΔC_S	ΔH_F	ΔH_M	ΔC_S: ΔH_F	ΔC_S: ΔH_M	B_F	B_M	B_F:E	B_M:E	R:E	G	R:G	R:B_F	R: B_M	k	logLik	AICc	ΔAICc	w_i	Ret.	Adj. w_i
A	-0.7						15.6							0.3	-0.1						-2.5		6	-22.7	60.1	7.8	0.0		
A	-1.2						19.1	0.0										-0.4	0.0				6	-22.8	60.4	8.1	0.0		
C	2.4						-8.8		-0.2	-0.3	0.4	-0.4	-0.2										8	-19.6	60.4	8.1	0.0		
C	2.8	-0.7	-0.1		0.1			0.0															6	-22.8	60.4	8.1	0.0		
A	1.1						10.5	0.0						-0.1				-0.2					6	-22.8	60.4	8.1	0.0		
C	0.0	0.8		0.2		-0.1		0.0															6	-22.9	60.5	8.2	0.0		
R	1.1						-4.1	0.0						0.1		0.0							6	-22.9	60.6	8.3	0.0		
A	-0.1						15.1	0.0							0.0			-0.3					6	-22.9	60.6	8.3	0.0		
A	1.5						4.4	NA						-0.2	0.3							-2.0	6	-23.0	60.7	8.4	0.0		
R	1.5						-5.6	0.0							0.3		0.0						6	-23.0	60.7	8.4	0.0		
A/R	3.3						-4.8	0.0						-0.1	0.0								6	-23.0	60.7	8.4	0.0		
R	1.5							0.0						-0.1	0.3		0.0						6	-23.0	60.8	8.5	0.0		
C	3.5						-8.1	0.0	-0.2		0.4		-0.3										7	-21.6	61.1	8.7	0.0		
R	-0.3							0.0						0.2	0.0	0.0							6	-23.1	61.1	8.8	0.0		
A	1.8						-0.9	0.0							0.1							-1.0	6	-23.2	61.1	8.8	0.0		
A	2.7						-10.0	0.0											0.0	0.2			6	-23.2	61.2	8.9	0.0		
C	2.0	-0.4	-0.1		0.1		-3.8																6	-23.3	61.5	9.2	0.0		
C	1.2							0.0	0.0	-0.3	0.4	-0.4	-0.2										8	-20.4	61.9	9.6	0.0		
C	0.9	0.8		0.2		-0.1	-5.9	0.0															7	-22.3	62.5	10.2	0.0		
C	0.7	-0.1	-0.3	0.2	0.1	-0.1																	7	-22.4	62.6	10.3	0.0		
A	-1.2						22.8	0.0						0.2				-0.2			-1.8		7	-22.5	62.9	10.6	0.0		
A	-1.0						19.8							0.2	0.2						-2.2	-1.5	7	-22.5	62.9	10.6	0.0		
A	0.2						13.2	0.0						0.3	-0.1						-2.2		7	-22.6	63.0	10.7	0.0		
R	2.2						-4.8	0.0						-0.1	0.3		0.0						7	-22.6	63.2	10.9	0.0		
C	3.2	-0.7	-0.1		0.1		-3.1	0.0															7	-22.7	63.2	10.9	0.0		
A	-0.3						12.4	0.0										-0.4	0.0	0.4			7	-22.7	63.3	11.0	0.0		
A	2.3						2.9	0.0						-0.1	0.2							-1.7	7	-22.8	63.5	11.1	0.0		
A	1.1						10.5	0.0						-0.1	0.0			-0.2					7	-22.8	63.5	11.2	0.0		
A	-0.7						19.3	0.0							0.1			-0.3				-1.0	7	-22.9	63.6	11.3	0.0		
R	1.1						-4.2	0.0						0.2	0.0	0.0							7	-22.9	63.6	11.3	0.0		
C	3.2						-9.0	0.0	-0.2	-0.3	0.5	-0.4	-0.2										9	-19.5	63.7	11.4	0.0		
R	0.4							0.0						0.0	0.3	0.0	0.0						7	-23.0	63.8	11.5	0.0		
C	1.9	-0.3	-0.2	0.2	0.2	-0.1		0.0															8	-21.9	64.8	12.5	0.0		
C	1.3	0.1	-0.3	0.3	0.1	-0.1	-4.9																8	-22.1	65.3	13.0	0.0		
A	-0.3						17.3	0.0						0.2	0.1						-2.0	-1.3	8	-22.5	66.1	13.8	0.0		
A	-1.2						22.9	0.0						0.3	0.0			-0.2			-2.0		8	-22.5	66.1	13.8	0.0		
R	2.2						-4.8	0.0						-0.1	0.3	0.0	0.0						8	-22.6	66.4	14.1	0.0		
A	0.5						15.5	0.0						-0.1	0.2			-0.2				-1.5	8	-22.7	66.5	14.2	0.0		
C	2.3	-0.2	-0.2	0.2	0.2	-0.1	-4.2	0.0															9	-21.6	67.9	15.6	0.0		
A	-1.5						25.9	0.0						0.2	0.1			-0.2			-1.9	-1.2	9	-22.4	69.5	17.2	0.0		

Hyp. = Hypothesis: A = Adaptive Forced Dispersal; C = Coercion of Cooperation; R = Reproductive Competition; Any = Any of the three hypotheses; Null = null model. Columns 2 to 23 show parameter effect sizes from GLMMs on the logit scale: Int. = Intercept; C_S = number of emergent pups per female that contributed to the previous litter; H_F = number of female helpers in the

previous breeding attempt; H_M = number of male helpers in the previous breeding attempt; R = mean group relatedness; E = mean rainfall in previous 6 months; ΔC_S = change in the number of emergent pups per female that contributed to the litter in the breeding attempts before and of the eviction; ΔH_F = change in the number of female helpers in the breeding attempts before and of the eviction; ΔH_M = change in the number of male helpers in the breeding attempts before and of the eviction; B_F = number of breeding females; B_M = number of breeding males; G = group size; : = interaction. k = number of estimated parameters including a random intercept for group ID; logLik = log-likelihood; AICc = corrected Akaike's information criterion; ΔAICc = change in AICc value from the best performing model; w_i = Akaike's model weight; Ret. = tick indicates that the model was retained after applying the nesting rule of Richards et al. (2011); Adj. w_i = adjusted Akaike's model weight for the retained models. Blank cells indicate that the term was absent from that model.

ESM Table 6: ‘Temporary evictions’. Model performance in predicting the probability that evictees are allowed to return to their group following an eviction event (N=22 eviction events in 6 groups). Analysis using the helping effort (C_E) measure of helping performance under the coercion of cooperation hypothesis. Models above the line comprise the top model set where ΔAICc ≤ 6.

Hyp.	Int.	C_{EF}	H_F	C_{EF}:H_F	C_{EM}	H_M	C_{EM}:H_M	R	E	ΔC_{EF}	ΔH_F	ΔC_{EF}:ΔH_F	ΔC_{EM}	ΔH_M	ΔC_E:ΔH_M	B_F	B_M	B_F:E	B_M:E	R:E	G	R:G	R:B_F	R:B_M	k	logLik	AICc	ΔAICc	w_i	Ret.	Adj. w_i
Null	0.0																								2	-15.2	35.1	0.0	0.3	✓	1.0
R	2.0															-0.2									3	-14.6	36.5	1.4	0.1		
R	0.9																-0.2								3	-14.7	36.8	1.7	0.1		
Any	-0.2							0.9																	3	-15.2	37.8	2.7	0.1		
C	-2.8	32.0	0.3	-4.0																					5	-12.1	38.0	2.9	0.1		
R	3.1															-0.2	-0.2								4	-14.0	38.4	3.2	0.0		
R	2.9								0.0								-0.1								4	-14.4	39.1	4.0	0.0		
R	3.1								0.0							-0.2									4	-14.4	39.2	4.0	0.0		
A/R	1.8							1.6								-0.2									4	-14.5	39.5	4.3	0.0		
Any	2.4							0.3	0.0																4	-14.7	39.8	4.7	0.0		
A/R	0.8							0.3									-0.2								4	-14.7	39.8	4.7	0.0		
A	0.9							0.7													0.0				4	-15.1	40.5	5.3	0.0		
C	0.3									3.5	-0.2	-1.0													5	-13.4	40.5	5.4	0.0		
A	-6.6							67.4	0.1											-1.0					5	-13.7	41.2	6.1	0.0		
A	-3.1							32.9								0.4							-4.1		5	-13.9	41.5	6.3	0.0		
C	-3.9	33.3	0.4	-4.2				4.7																	6	-12.0	41.5	6.4	0.0		
R	3.8								0.0							-0.2	-0.2								5	-13.9	41.6	6.5	0.0		
R	23.3								-0.3							-2.5		0.0							5	-14.0	41.7	6.6	0.0		
A/R	3.0							0.9								-0.2	-0.2								5	-14.0	41.8	6.6	0.0		
C	-2.0	31.4	0.4	-4.0					0.0																6	-12.1	41.8	6.7	0.0		
R	3.4								0.0								-0.2		0.0						5	-14.4	42.5	7.4	0.0		
A/R	3.0							-0.2	0.0								-0.1								5	-14.4	42.5	7.4	0.0		
A/R	2.9							1.0	0.0							-0.2									5	-14.4	42.5	7.4	0.0		
C	3.7								-0.1	3.5	0.0	-1.0													6	-12.7	42.9	7.8	0.0		
A	1.8							-5.9									-0.4							1.5	5	-14.7	43.1	8.0	0.0		
A	2.4							0.3	0.0												0.0				5	-14.7	43.2	8.1	0.0		
A	-3.6							46.5								0.7	-0.3						-5.9		6	-12.9	43.3	8.2	0.0		
C	1.1				-0.3	-0.1	0.1																		5	-14.8	43.4	8.2	0.0		
C	-0.1												-0.3	0.1	0.0										5	-15.0	43.8	8.6	0.0		
A	0.4							4.3													0.0	-0.2			5	-15.0	43.8	8.7	0.0		
A	-7.3							79.6	0.1								-0.2			-1.2					6	-13.1	43.9	8.8	0.0		
C	0.9							-2.9		3.9	-0.2	-1.2													6	-13.3	44.2	9.1	0.0		
A	-5.4							61.8	0.1							-0.1				-0.9					6	-13.5	44.7	9.5	0.0		
A	-2.8							44.3	0.0							0.8							-6.0		6	-13.6	44.7	9.6	0.0		
R	19.0								-0.3							-2.0	-0.1	0.0							6	-13.7	45.0	9.9	0.0		
A	-6.6							67.6	0.1											-1.0	0.0				6	-13.7	45.0	9.9	0.0		
R	24.5							2.7	-0.4							-2.8		0.0							6	-13.9	45.4	10.3	0.0		
C	4.3								-0.1				-0.7	0.3	0.5										6	-13.9	45.5	10.3	0.0		
A/R	3.7							0.5	0.0							-0.2	-0.2								6	-13.9	45.5	10.3	0.0		
R	3.5								0.0							-0.2	-0.1		0.0						6	-13.9	45.5	10.3	0.0		

Hyp.	Int.	C_{EF}	H_F	C_{EF}: H_F	C_{EM}	H_M	C_{EM}: H_M	R	E	ΔC_{EF}	ΔH_F	ΔC_{EF}: ΔH_F	ΔC_{EM}	ΔH_M	ΔC_E: ΔH_M	B_F	B_M	B_F:E	B_M:E	R:E	G	R:G	R:B_F	R:B_M	k	logLik	AICc	ΔAICc	w_i	Ret.	Adj. w_i
A	3.1							-0.1								-0.2	-0.2							0.2	6	-14.0	45.6	10.5	0.0		
C	-3.1	32.9	0.4	-4.1				4.6	0.0																7	-11.9	45.9	10.7	0.0		
C	3.5				-0.3	-0.1	0.1		0.0																6	-14.3	46.1	11.0	0.0		
A	3.4							-3.4	0.0								-0.3							0.8	6	-14.4	46.3	11.2	0.0		
R	3.4							-0.1	0.0								-0.2		0.0						6	-14.4	46.3	11.2	0.0		
A	1.5							7.9	0.0												0.1	-0.4			6	-14.7	47.0	11.8	0.0		
C	0.8				-0.4	-0.2	0.1	2.5																	6	-14.7	47.1	11.9	0.0		
C	4.2							-3.1	-0.1	4.0	-0.1	-1.3													7	-12.6	47.1	12.0	0.0		
A	-2.7							46.6	0.0							0.8	-0.3						-6.1		7	-12.7	47.5	12.3	0.0		
C	-0.2							0.8					-0.3	0.1	0.0										6	-15.0	47.6	12.5	0.0		
A	-3.3							44.5								0.8	-0.5						-6.4	1.5	7	-12.8	47.6	12.5	0.0		
A	-5.9							73.0	0.1							-0.2	-0.2			-1.1					7	-12.9	47.8	12.7	0.0		
A	-6.5							70.8	0.1								-0.6			-1.3				2.6	7	-13.0	48.0	12.9	0.0		
A	-6.6							72.0	0.1							0.5				-0.7			-3.8		7	-13.3	48.5	13.4	0.0		
A	-7.2							70.2	0.2											-1.5	-0.2	1.2			7	-13.5	49.0	13.9	0.0		
R	19.9							1.9	-0.3							-2.2	-0.1	0.0							7	-13.7	49.3	14.2	0.0		
C	-1.5	65.7	0.9	-8.2	-4.7	-0.4	0.3																		8	-11.1	49.3	14.2	0.0		
R	21.0								-0.3							-2.1	-0.3	0.0	0.0						7	-13.7	49.4	14.2	0.0		
C	4.2							0.2	-0.1				-0.7	0.3	0.5										7	-13.9	49.9	14.7	0.0		
R	3.5							0.4	0.0							-0.2	-0.1		0.0						7	-13.9	49.9	14.7	0.0		
A	3.6							0.6	0.0							-0.2	-0.2							0.0	7	-13.9	49.9	14.7	0.0		
C	3.3				-0.4	-0.1	0.1	2.0	0.0																7	-14.2	50.5	15.3	0.0		
A	-6.5							75.3	0.1							0.5	-0.3			-0.7			-4.2		8	-12.4	51.9	16.8	0.0		
A	-2.6							44.9	0.0							0.8	-0.4						-6.3	1.0	8	-12.7	52.5	17.4	0.0		
A	-5.5							67.7	0.1							-0.1	-0.5			-1.1				1.7	8	-12.9	52.8	17.7	0.0		
C	-4.1	103.6	1.6	-13.0	-7.7	-0.8	0.5	10.2																	9	-10.7	54.3	19.2	0.0		
R	23.0							2.3	-0.3							-2.4	-0.4	0.0	0.0						8	-13.6	54.4	19.2	0.0		
C	-3.8	88.7	1.3	-11.1	-6.7	-0.7	0.4		0.0																9	-11.0	54.9	19.8	0.0		
A	-6.4							72.4	0.1							0.5	-0.5			-0.7			-4.4	1.9	9	-12.4	57.8	22.6	0.0		
C	-7.7	135.9	2.1	-17.1	-10.5	-1.1	0.7	11.0	0.1																10	-10.4	60.8	25.7	0.0		

Hyp. = Hypothesis: A = Adaptive Forced Dispersal; C = Coercion of Cooperation; R = Reproductive Competition; Any = Any of the three hypotheses; Null = null model. Columns 2 to 25 show parameter effect sizes from GLMMs on the logit scale: Int. = Intercept; C_{EF} = number of female helpers left per day of babysitting of the previous litter; H_F = number of female helpers in the previous breeding attempt; C_{EM} = number of male helpers left per day of babysitting of the previous litter; H_M = number of male helpers in the previous breeding attempt; R = mean group relatedness; E = mean rainfall in previous 6 months; ΔC_{EF} = change in the number of female helpers left per day of babysitting in the breeding attempts before and of the eviction; ΔH_F = change in the number of female helpers in the breeding attempts before and of the eviction; ΔC_{EM} = change in the number of male helpers left per day of babysitting in the breeding attempts before and of the eviction; ΔH_M = change in the number of male helpers in the breeding attempts before and of the eviction; B_F = number of breeding females; B_M = number of breeding males; G = group size; : = interaction. k = number of estimated parameters including a random intercept for group ID; logLik = log-likelihood; AICc = corrected Akaike's information criterion;

ΔAICc = change in AICc value from the best performing model; w_i = Akaike’s model weight; Ret. = tick indicates that the model was retained after applying the nesting rule of Richards et al. (2011); Adj. w_i = adjusted Akaike’s model weight for the retained models. Blank cells indicate that the term was absent from that model.
